# The Rice Small Auxin-Up RNA Gene *OsSAUR33* Regulates Seed Vigor via Sugar Pathway during Early Seed Germination

**DOI:** 10.3390/ijms22041562

**Published:** 2021-02-04

**Authors:** Jia Zhao, Wenjun Li, Shan Sun, Liling Peng, Zhibo Huang, Yongqi He, Zhoufei Wang

**Affiliations:** The Laboratory of Seed Science and Technology, Guangdong Key Laboratory of Plant Molecular Breeding, Guangdong Laboratory of Lingnan Modern Agriculture, State Key Laboratory for Conservation and Utilization of Subtropical Agro-Bioresources, South China Agricultural University, Guangzhou 510642, China; jiazhao@scau.edu.cn (J.Z.); lwj2019@stu.scau.edu.cn (W.L.); 20192015015@stu.scau.edu.cn (S.S.); 20192040003@stu.scau.edu.cn (L.P.); 20202015013@stu.scau.edu.cn (Z.H.)

**Keywords:** rice, seed vigor, small auxin-up RNA, sugar pathway

## Abstract

Seed vigor affects seed germination and seedling emergence, and therefore is an important agronomic trait in rice. Small auxin-up RNAs (*SAURs*) function in a range of developmental processes, but their role in seed vigor remains unclear. Here, we observed that disruption of *OsSAUR33* resulted in reduced germination rates and low seed uniformity in early germination. Expression of *OsSAUR33* was higher in mature grains and early germinating seeds. RNA-seq analysis revealed that *OsSAUR33* modulated seed vigor by affecting the mobilization of stored reserves during germination. Disruption of *OsSAUR33* increased the soluble sugar content in dry mature grains and seeds during early germination. OsSAUR33 interacted with the sucrose non-fermenting-1-related protein kinase OsSnRK1A, a regulator of the sugar signaling pathway, which influences the expression of sugar signaling-related genes during germination. Disruption of *OsSAUR33* increased sugar-sensitive phenotypes in early germination, suggesting OsSAUR33 likely affects seed vigor through the sugar pathway. One elite haplotype of *OsSAUR33* associated with higher seed vigor was identified mainly in *indica* accessions. This study provides insight into the effects of OsSAUR33 on seed vigor in rice.

## 1. Introduction

Rice (*Oryza sativa* L.) is one of the most important food crops in the world. Direct seeding of rice has become popular in China and South Asia over the past three decades due to its low cost and operational simplicity [1,2]. However, there are some issues with direct seeding compared with transplanting, such as poor seedling establishment and challenges with weed competition. Seeds with high vigor have rapid germination, good seedling establishment, and vigorous seedling growth [3,4]. Good seedling establishment is very important in direct seeding of rice for establishing sufficient numbers of plants and thus for yield [5]. Therefore, cloning seed vigor-related genes in rice and elucidating their molecular mechanisms may have applications in breeding programs.

Seed germination and seedling growth require large amounts of energy and nutrition. In rice, the starchy endosperm makes up the largest proportion of grain dry weight and provides the major carbon source for generating energy and metabolites during seed germination. The amylose and amylopectin in the native starch granule are first hydrolyzed by α-amylase, and then the released oligosaccharides are further hydrolyzed by α-amylase until glucose and maltose are produced [6]. The expression of α-amylase genes is activated by the hormone gibberellic acid (GA) in the endosperm [7]. The transcription factor Gibberellin MYB gene (MYBGA) is a GA-inducible R2R3 MYB that binds to the GA-responsive element (GARE) and activates the promoters of α-amylases and other hydrolases in cereal aleurone cells [8,9]. GA-induced DELLA protein degradation is another central regulatory system in the GA signaling pathway [10]. Thus, MYBGA and DELLA are important factors in the GA signaling pathway during seed germination. Additionally, abscisic acid (ABA) and auxin (indole-3-acetic acid, IAA) play essential roles in the inhibition of seed germination through the regulation of *ABA-INSENSITIVE3/5* expression [11,12].

The expression of α-amylase genes is also activated by sugar starvation in embryos [7,13], but it is repressed by the presence of almost all metabolizable sugars [14,15,16]. Upon imbibition of cereal grains, sugars in the embryo are rapidly consumed, leading to sugar starvation for the activation of α-amylase synthesis in the scutellum [17,18]. The promoters of α-amylase genes are activated by sugar starvation through the sugar response complex (SRC), via the TA box sequence motif, a key sugar response element [7,13,19]. Rice MYBS1 is a sugar-repressible R1 MYB transcription factor that contains a single DNA binding repeat and binds specifically to the TA box, where it acts as a transcriptional activator in the *aAmy3* SRC under sugar-depleted conditions [20,21]. Recently, the roles of MYBS1 and MYBS2 under sugar starvation in rice were revealed; MYBS1 promotes *αAmy* expression whereas MYBS2 represses it, providing an on/off switch for *αAmy* expression [22].

The plant sucrose non-fermenting 1 (SNF1)-related protein kinases (SnRKs), which are Ser/Thr protein kinases, are grouped into three subfamilies (SnRK1, SnRK2, and SnRK3) and are involved in various physiological processes [23]. In rice, the *SnRK1* gene family has two members, *SnRK1A* and *SnRK1B*, in which *SnRK1A* is uniformly expressed in various growing tissues, including young roots and shoots, flowers, and immature seeds [24]. *SnRK1A* plays important roles in the regulation of seed germination and seedling growth in rice. For example, rice *SnRK1A* acts upstream of *MYBS1* and *aAmy3* and plays a central role in the sugar signaling pathway by regulating *MYBS1* and *aAmy3* expression during seed germination and seedling growth [21]. Meanwhile, rice *SnRK1A* acts as an important regulator for seed germination and seedling growth under hypoxic conditions [25]. Moreover, *SnRK1A* plays a key role in regulating source–sink communication during seedling growth in rice [26]. Taken together, these observations show that rice *SnRK1A* has very important roles in sugar signaling, stress tolerance, seed germination, and seedling growth.

Plant *AUX/IAAs*, *GRETCHEN HAGEN3s* (*GH3s*), and *SMALL AUXIN UP RNAs* (*SAURs*) are three families of early auxin response genes [27]. Among them, the *SAUR* gene family is the most numerous in plants, with 81 *SAURs* in *Arabidopsis thaliana* [27] and 58 *SAURs* in rice [28], which are involved in a wide range of cellular, physiological, and developmental processes [29]. For example, *SAUR36* [30], *SAUR41* [31], *SAUR19* [32], and *SAUR63* [33] positively regulate cell expansion to promote hypocotyl growth in *Arabidopsis*. The overexpression lines of *Arabidopsis SAUR76* and rice *OsSAUR39* display enhanced and inhibited root growth, respectively [34]. SAUR proteins also modulate the phosphorylation status of plasma membrane H^+^-ATPases to regulate cell expansion by inhibiting the activity of a family of type 2C protein phosphatases (PP2Cs) in shoot growth of *Arabidopsis* [35]. However, the underlying roles of *SAURs* in the regulation of seed vigor remain unclear in rice.

Our previous RNA-seq data showed that the expression of *OsSAUR33* was significantly induced at the early seed germination stage in rice, suggesting that it might be involved in the regulation of seed vigor [36]. In this study, we investigated the regulatory functions of *OsSAUR33* in seed vigor. Disruption of *OsSAUR33* resulted in low seed vigor at the early germination stage in rice. We observed that OsSAUR33 interacts with SnRK1A in rice. Our results suggest that *OsSAUR33* acts in the regulation of seed vigor through the sugar pathway in early seed germination. The application of *OsSAUR33* will be useful in rice breeding programs for improvement of rice seed vigor for direct seeding cultivation.

## 2. Results

### 2.1. Disruption of *OsSAUR33* Results in Low Seed Vigor

We identified 48 genes encoding OsSAUR proteins with a typical auxin-inducible domain (pfam02519) in the rice genome (http://rice.plantbiology.msu.edu/; Table S1). These *OsSAURs* can be divided into two classes of 24 members each (Figure S1A). Further genevestigator (https://genevestigator.com/) analysis showed that *OsSAUR33* was expressed at high levels in both embryo and endosperm tissues during seed germination (Figure S1B,C). This suggests that *OsSAUR33* might affect seed vigor based on our previous study [36] in rice. To test this, we employed the clustered regularly interspaced short palindromic repeats (CRISPR)/CRISPR-associated protein 9 (Cas9) genome editing system to generate mutants, which were named *ossaur33-1* and *ossaur33-2* (Figure S2A,B). The sequence changes of the edited targets caused premature termination of the *ossaur33-1* and *ossaur33-2* mutant transcripts (Figure S2C,D). The progeny of these homozygous mutants was used in subsequent experiments.

**Figure 1:**
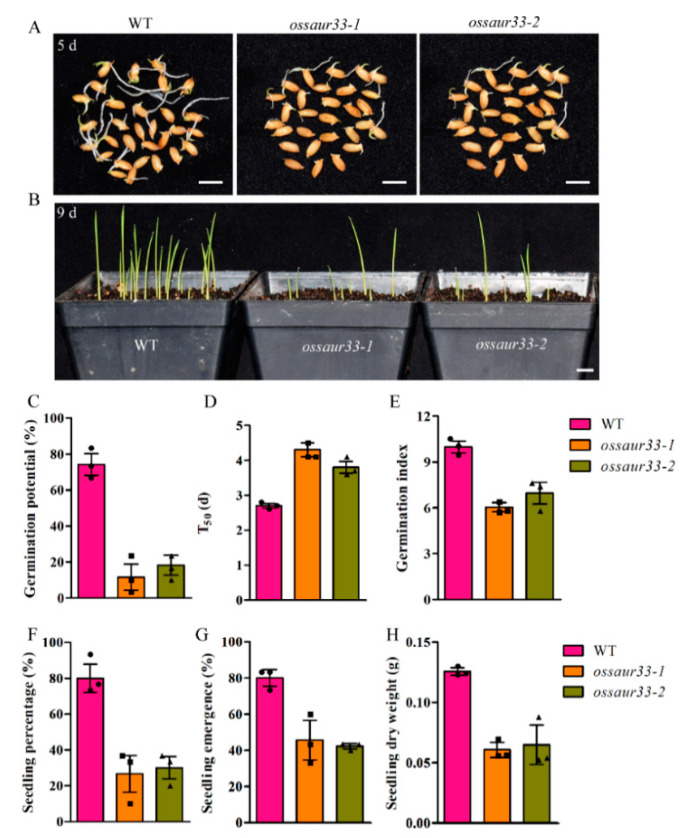
Comparison of seed vigor between the wild type (WT) and the *ossaur33* mutants in rice. (**A**) Seed germination of the WT and *ossaur33* mutants after 5 days. (**B**) Seedling establishment of the WT and *ossaur33* mutants 9 days after direct seeding in soils. Bar = 10 mm. The seed vigor traits, including (**C**) germination potential, (**D**) T_50_, time to 50% germination, (**E**) germination index, and (**F**) seedling percentage, under normal conditions, and (**G**) seedling emergence, and (**H**) seedling dry weight after direct seeding. Each column represents the mean ± standard deviation, black small symbol means the value of each replication, *n* = 3.

The disruption of *OsSAUR33* resulted in low seed vigor at the germination stage in rice. Germination speed and uniformity, including germination potential (the germination percentage after 3 days), germination index (the sum of the day’s germinated grain number/germination days during 9 days germination stage), and seedling percentage (the percentage of seedling establishment), were decreased in the *ossaur33* mutant lines, while the T_50_ (the time to reach 50% germination) was increased compared to the wild-type (WT) Nipponbare plants (Figure 1A–F). For example, less than 20% of *ossaur33* seeds germinated after 5 days, in contrast to 75% of WT seeds. Meanwhile, seedling emergence, and the seedling dry weight were significantly decreased in *ossaur33* mutant lines compared to the WT plants after direct seeding in soils (Figure 1G,H). The percentage of emerged seedlings for *ossaur33* lines was approximately 45% at 9 days after direct seeding while it was 80% in the WT. This suggests that *OsSAUR33* plays important regulatory roles in germination speed and uniformity, and seedling growth in rice.

### 2.2. Expression Patterns of *OsSAUR33* and Subcellular Localization

**Figure 2:**
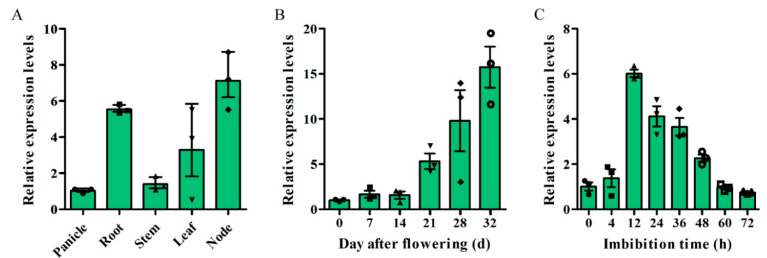
Expression patterns of *OsSAUR33* by quantitative reverse transcription polymerase chain reaction (qRT-PCR) in rice. (**A**) Expression pattern of *OsSAUR33* in various tissues, including panicle, root, stem, leaf, and internode, of rice. Expression pattern of *OsSAUR33* in various developmental stages from 0 to 32 days after flowering (**B**) and during seed germination from 0 to 72 h of imbibition (**C**) in rice. The expression of *OsSAUR33* was normalized to that of *OsActin*. Each column represents the mean ± standard deviation, black small symbol means the value of each replication, *n* = 3.

In order to further clarify the physiological function of *OsSAUR33*, we analyzed its expression in various tissues and in developing and germinating seeds of rice using quantitative reverse transcription polymerase chain reaction (qRT-PCR). Relatively higher expression of *OsSAUR33* was observed in the root and internode compared with that in the panicle, stem, and leaf (Figure 2A). The expression of *OsSAUR33* gradually increased in the filling grains (0 to 32 days after flowering), and it reached the highest level at the seed maturity stage (Figure 2B). During seed germination, the transcript level of *OsSAUR33* first increased and then decreased with the increase of imbibition time (0 to 72 h), peaking at 12 h of imbibition (Figure 2C).

**Figure 3:**
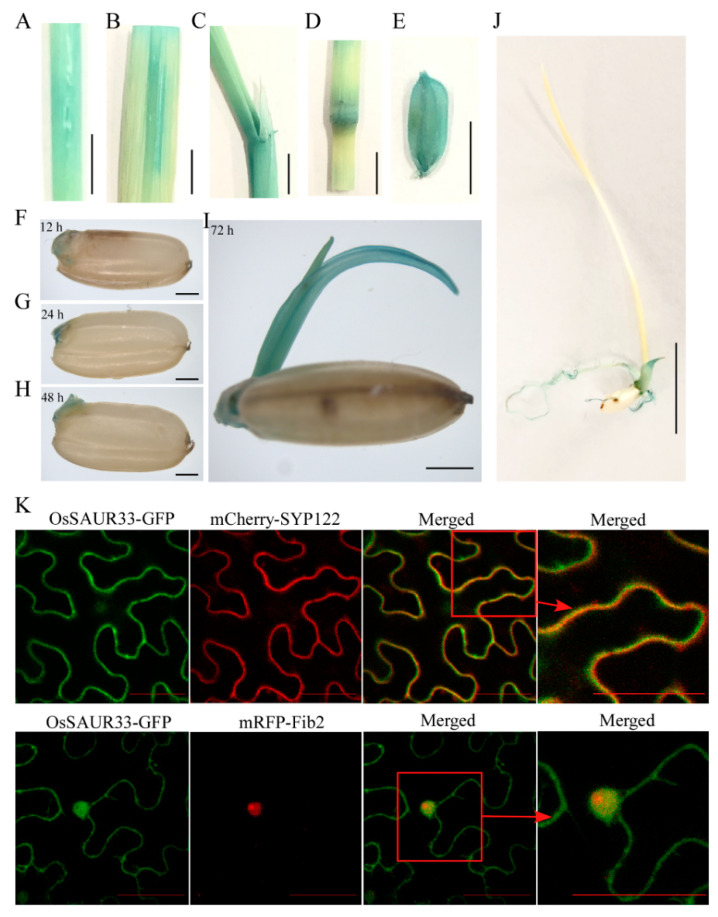
β-Glucuronidase (GUS) activity of the *OsSAUR33* promoter and its subcellular localization in rice. (**A**–**J**) Histochemical staining for GUS activity in various tissues and germinating seeds. Bar = 1 cm (**K**) Subcellular localization of OsSAUR33 in *N. benthamiana* leaves. The localization of mCherry-SYP122, a plasma membrane (PM) marker, and mRFP-Fib2, a nucleus marker, is shown in red, and OsSAUR33 fusion proteins is shown in green. The red box indicates the higher magnification image of localization. Fluorescence signals were observed at 48 h after infection. Bar = 50 μm.

To further investigate the tissue-specific expression of *OsSAUR33*, we performed histochemical staining for β-glucuronidase (GUS) activity of the *OsSAUR33* promoter:*GUS* transgenic lines in rice. GUS was strongly expressed in leaf, stem, internode, root, and panicle tissues, as well as in the germinating embryos and shoots (Figure 3A–J). This finding is consistent with the above qRT-PCR results. To determine the subcellular localization of OsSAUR33, we constructed a recombinant OsSAUR33 protein tagged at the C terminus with green fluorescent protein (GFP) under the control of the 35S promoter and expressed it transiently in *Nicotiana benthamiana* leaves. Confocal microscopy revealed that OsSAUR33-GFP signals co-localized with the red fluorescence signal of mCherry-SYP122, a plasma membrane (PM) marker, and of mRFP-Fib2, a nucleus marker, respectively, indicating that OsSAUR33 was likely localized in the plasma membrane and nucleus (Figure 3K).

### 2.3. Disruption of *OsSAUR33* Alters the Sugar Level in Mature Grains and in Seeds at the Early Germination Stage

**Figure 4:**
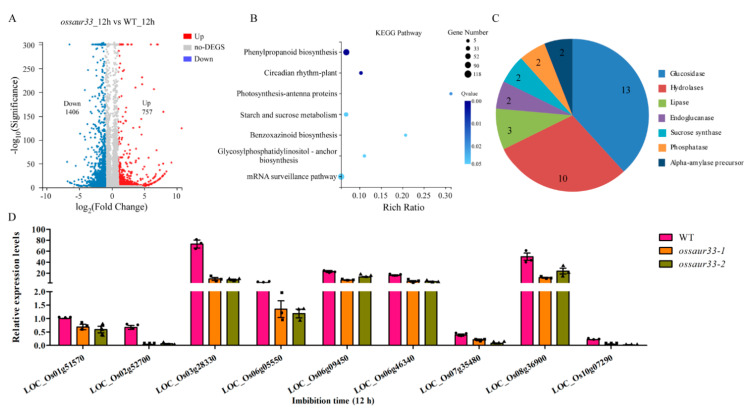
Rice *OsSAUR33* alters the mobilization of stored reserves during seed germination. (**A**) The differentially expressed genes (DEGs) with *p* < 0.001 and fold change ≥ 2 between the WT and the *ossaur33-1* mutant in the early (12 h) germinating seeds. Red, up-regulation; Gray, no change; Blue, down-regulation. (**B**) Kyoto Encyclopedia of Genes and Genomes (KEGG) analysis of the DEGs. (**C**) DEGs involved in starch and sucrose metabolism. (**D**) The expression confirmation of starch and sucrose metabolism-related DEGs in the early (12 h) germinating seeds using qRT-PCR. The expression of genes was normalized to that of *OsActin*. Each column represents the mean ± standard deviation, black small symbol means the value of each replication, *n* = 3.

To further understand the function of *OsSAUR33* in regulating seed vigor, we compared genome-wide transcript levels between *ossaur33-1* and WT germinating seeds at 12 h of imbibition due to the high expression observed at that stage in our qRT-PCR analysis. A total of 2163 differentially expressed genes (DEGs) with at least a 2-fold change (*p* < 0.001) were identified between the *ossaur33-1* mutant and the WT. Of these, 1406 were down-regulated in the mutant, and 757 were up-regulated (Figure 4A, Table S2). Kyoto Encyclopedia of Genes and Genomes (KEGG) analysis showed that the DEGs were significantly enriched in starch and sucrose metabolism (Figure 4B). Among them, the majority belonged to glucosidase (13) and hydrolase (10)-related genes (Figure 4C, Table S3). As expected, our qRT-PCR analysis showed that the expression of these glucosidase- and hydrolase-related genes was down-regulated in the *ossaur33* mutants compared to the WT after 12 h of imbibition (Figure 4D). These results suggested that *OsSAUR33* may regulate seed vigor by promoting the mobilization of stored reserves during seed germination in rice.

Gene expression analysis indicated that *OsSAUR33* was highly expressed in the late developing grains, suggesting that it may affect seed quality in rice. Seed quality is established during seed development and affects seed vigor during germination. We observed that the grain size, including grain length, width, and thickness, and 1000-grain weight, as well as plant height, heading date, number of tillers/plant, and number of grains/main panicle, were not influenced by the loss of *OsSAUR33* (Figure S3).

**Figure 5:**
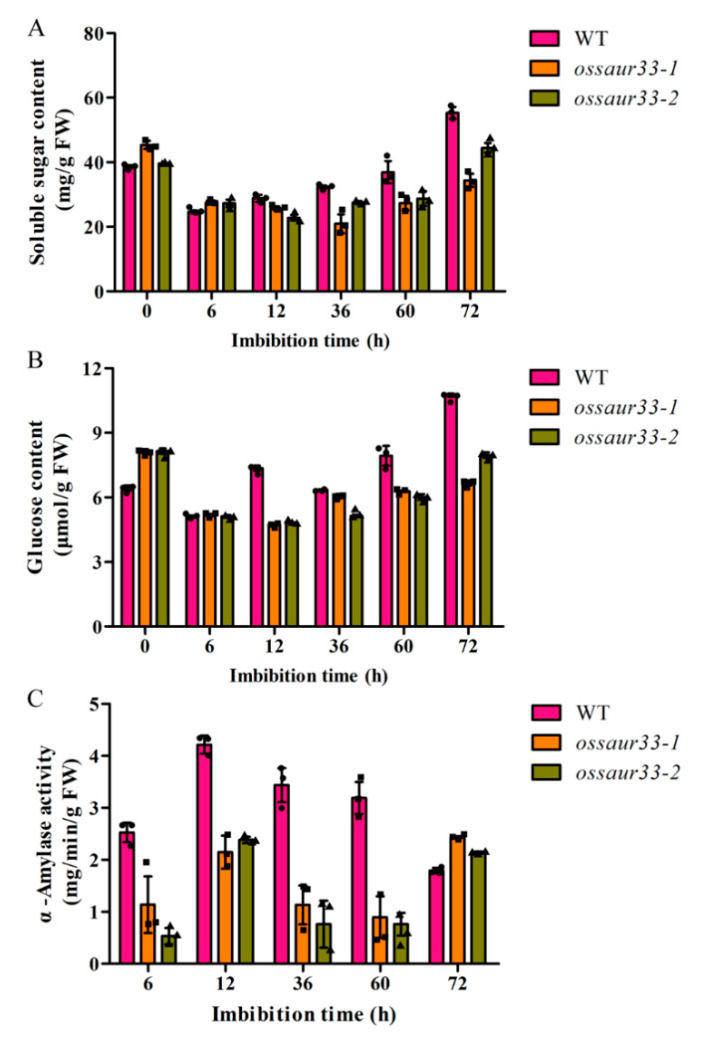
*OsSAUR33* regulates sugar levels during seed germination in rice. (**A**) Total soluble sugar; (**B**) Glucose; (**C**) α-amylase activity. Each column represents the mean ± standard deviation, black small symbol means the value of each replication, *n* = 3.

Our RNA-seq data suggested that the mechanism of OsSAUR33-mediated regulation of seed vigor might involve the sugar levels in seeds. Thus, the sugar contents in the mature grains and germinating seeds were compared between the *ossaur33* mutants and the WT. The total soluble sugar and glucose contents were higher in the *ossaur33* mutant seeds compared to those of the WT at 0 h (dry mature seed) and 6 h of imbibition, while the contents were lower at 12, 36, 60, and 72 h of imbibition (Figure 5A,B). Additionally, the lower α-amylase activities were observed in the *ossaur33* mutants at 6 to 60 h of imbibition but higher activities were observed at 72 h of imbibition compared with the WT (Figure 5C). These results suggest that OsSAUR33 regulates starch mobilization by modulating α-amylase hydrolase activity during seed germination in rice.

### 2.4. Sugar Pathway is Involved in *OsSAUR33*-Mediated Regulation of Seed Vigor

**Figure 6:**
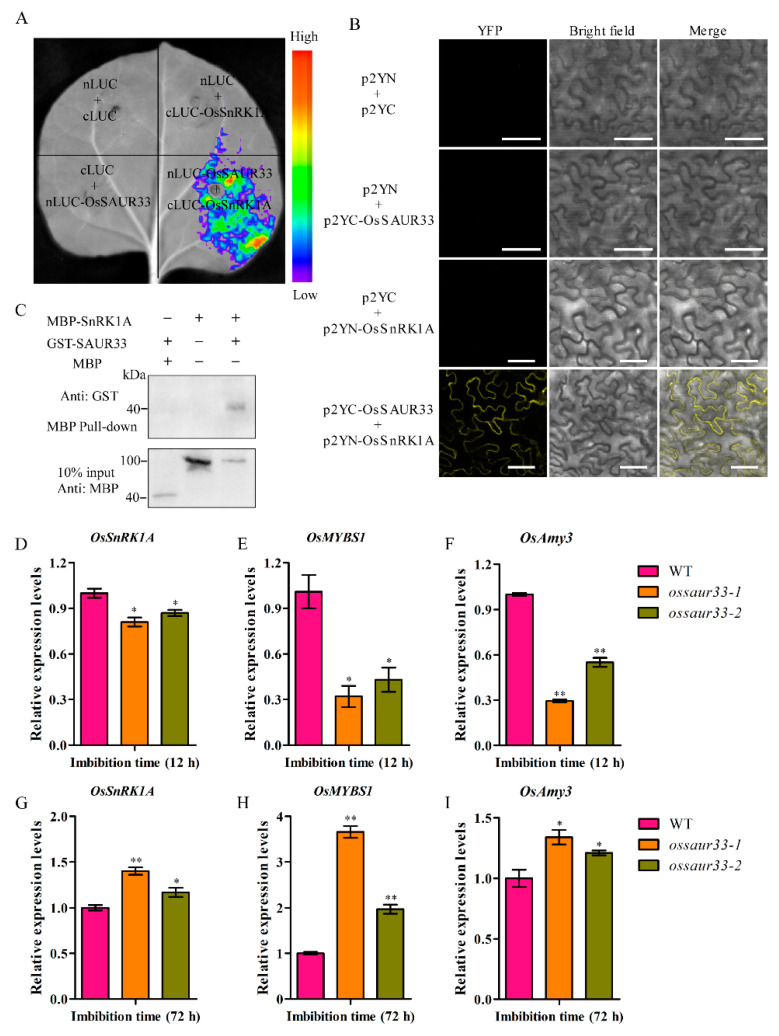
OsSAUR33 interacts with OsSnRK1A and alters the expression of *OsSnRK1A*, *OsMYBS1*, and *OsAmy3* during seed germination in rice. (**A**) Firefly luciferase (LUC) complementation imaging assay. nLUC-OsSnRK1A and cLUC-OsSAUR33 with the control vector were co-infiltrated into *N. benthamiana* leaves. LUC images were captured using a cooled Charge-coupled Device (CCD) imaging apparatus. (**B**) Bimolecular fluorescence complementation (BiFC) assay. OsSAUR33 and OsSnRK1A were fused to p2YC and p2YN, respectively. Different pairs of constructs were co-expressed in *N. benthamiana*. Yellow fluorescent protein (YFP) fluorescence was detected by confocal microscopy. Bar = 50 μm. (**C**) Maltose Binding Protein (MPB) pull-down assay of the OsSAUR33 and OsSnRK1A interaction. Anti-GST antibody was used to detect the output protein. The expression of *OsSnRK1A* (**D**,**G**), *OsMYBS1* (**E**,**H**), and *OsAmy3* (**F**,**I**) in the WT and the *ossaur33* mutants at 12 h and 72 h of imbibition. The expression of genes was normalized to that of *OsActin*. Each column represents the mean ± standard deviation, black small symbol means the value of each replication, *n* = 3.

To determine the underlying mechanism by which OsSAUR33 affects seed vigor, we performed a yeast two-hybrid (Y2H) assay using OsSAUR33 as a bait to screen a cDNA library. OsSnRK1A was identified as a candidate interactor. OsSnRK1A is an important intermediate in the sugar signaling cascade and plays a key role in regulating seed germination and seedling growth in rice [21]. We then confirmed the OsSAUR33–OsSnRK1A interaction by luciferase (LUC) and bimolecular fluorescence complementation (BiFC) assays. Our results showed that only co-expression of nLUC-OsSnRK1A and cLUC-OsSAUR33 in tobacco leaves could reconstitute LUC activity compared with the various negative controls (Figure 6A). Meanwhile, the yellow fluorescent protein (YFP) signals were only observed on the plasma membrane of *N. benthamiana* leaves when p2YC-OsSAUR33 was co-infiltrated with p2YN-OsSnRK1A but not with the control constructs (Figure 6B). In order to further confirm the in vitro interaction between OsSAUR33 and OsSnRK1A, a Maltose Binding Protein (MBP) pull-down assay was employed. MBP-SnRK1A was used to pull down GST-OsSAUR33, which were successfully detected by an anti-GST antibody (Figure 6C; Figure S4). Rice *SnRK1A* was shown to act upstream of *MYBS1* and *aAmy3* expression during seed germination [21]. These results demonstrated that OsSAUR33 interacts with OsSnRK1A, and OsSAUR33 regulates α-amylase activities via the sugar signaling pathway.

The repression of α-amylase gene expression by sugar has been well studied in rice—[14,15,16,21,22]. *OsMYBS1* expression is repressed by sugars, and OsMYBS1 promotes *aAmy3* expression, which is an essential component of the sugar signaling pathway during seed germination in rice [20,21]. Therefore, the expression of *OsSnRK1A*, *OsMYBS1*, and *aAmy3* was further analyzed at early (12 h) and later (72 h) germination stages. The transcript levels of *OsSnRK1A*, *OsMYBS1*, and *OsAmy3* were reduced in the *ossaur33* mutants compared with the WT at the early germination stage, possibly due to the higher sugar contents in the mutants (Figure 6D–F). However, at the later germination stage, higher *OsSnRK1A*, *OsMYBS1*, and *OsAmy3* expression was observed in the *ossaur33* mutants, likely due to the lower sugar contents (Figure 6G–I). This is consistent with the above results that α-amylase activity was reduced in the *ossaur33* mutants compared with that in the WT at the early germination stage but was higher at the later germination stage.

**Figure 7:**
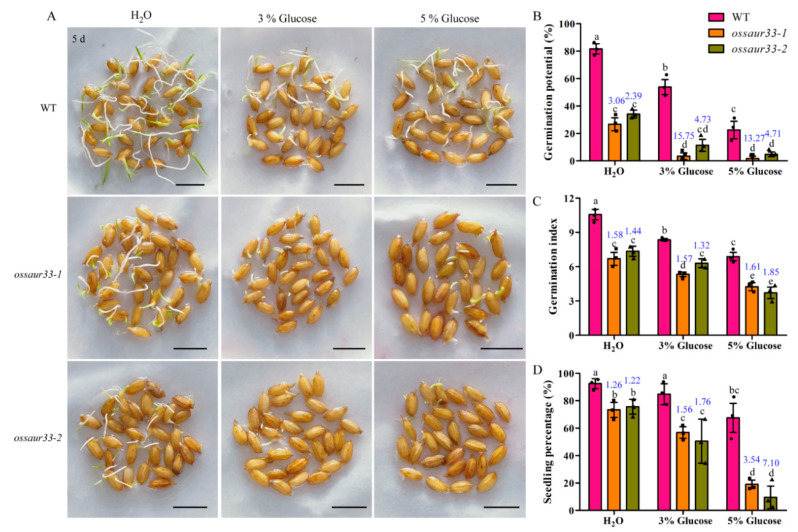
*OsSAUR33* regulates seed vigor via in the sugar pathway during early seed germination in rice. (**A**) Seed germination of the WT and the *ossaur33* mutants under H_2_O and glucose (3 and 5%) treatments for 5 days. Bars = 10 mm. (**B**–**D**) Comparison of the germination potential, germination index, and seedling percentage between the WT and *ossaur33* mutants under the H_2_O and glucose treatments conditions. The numbers above the box-plots indicate the relative value of the WT compared with that of the mutant. Each column represents the mean ± standard deviation, black small symbol means the value of each replication, *n* = 3. Different letters above the column indicate significant difference at the 5% level according to an analysis of variance (ANOVA) test.

Therefore, we speculated that the higher soluble sugar content, especially in dry mature seeds of the *osasur33* mutants, might cause low seed vigor. To confirm this, we analyzed the impact of various concentrations of exogenous glucose (3 and 5%) on the vigor of *osasur33* and WT seeds. In the presence of exogenous glucose, the *osasur33* mutants exhibited a sugar-sensitive phenotype with the significantly lower seed vigor especially in seedling percentage trait compared with that of the WT seeds (Figure 7A). By comparison, the relative suppression of seed vigor (i.e., the ratio WT/mutant), including the relative germination potential, germination index, and seedling percentage, was significantly greater under exogenous glucose treatment than in the control (treated with water only; Figure 7B–D). Therefore, we predicted that the higher soluble sugar content in mature seeds and in the early germinating seeds of the *ossaur33* mutants explains their low seed vigor.

### 2.5. Natural Variation in *OsSAUR33* is Associated with Seed Vigor in Rice

**Figure 8:**
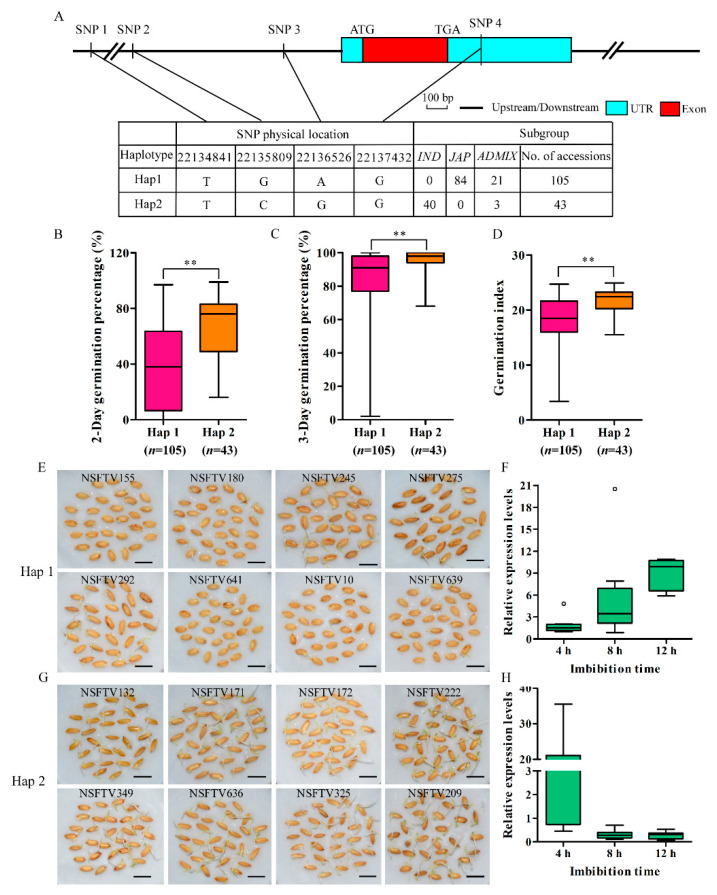
Haplotypes of *OsSAUR33* associated with seed vigor in rice. (**A**) Haplotypes of *OsSAUR33* identified in the region from ~2 kb upstream of the gene and its coding region. (**B**–**D**) Comparison of the germination percentage and germination index between accessions harboring different haplotypes. The number of rice accessions is listed in brackets. ** indicates significant difference at the 1% level according to Student’s *t*-test. (**E**,**G**) Seed germination (after 3 days) of accessions harboring different haplotypes. Bars = 10 mm. (**F**,**H**) Relative expression levels of *OsSAUR33* in rice accessions harboring different haplotypes during seed germination using qRT-PCR. The expression of genes was normalized to that of *OsActin*. Box plots represent the interquartile range, the thick line in the middle of each box represents the median, the whiskers represent 1.5 times the interquartile range, and the black circles represent outlier points.

To investigate whether the variation in different *OsSAUR33* alleles is associated with differences in seed vigor, we analyzed the single-nucleotide polymorphisms (SNPs) in the region from ~2 kb upstream of *OsSAUR33* and its coding region using the SNP data of 180 rice accessions (Table S4) [37]. Two haplotypes of *OsSAUR33* were identified among these accessions (Figure 8A). The elite haplotype, Hap 2, associated with high seed vigor and mainly existed in *indica* accessions; by contrast, Hap 1 associated with low seed vigor, mainly existed in *japonica* accessions (Figure 8B–D). Several elite *indica* accessions harboring Hap 2 were identified with high seed vigor (germination percentage after 2 days of greater than 75%), and several *japonica* accessions harboring Hap 1 were identified with low seed vigor (germination percentage after 2 days of less than 15%) (Figure 8E,G, Table S5). The expression of *OsSAUR33* in the accessions was analyzed during seed germination (0 to 12 h of imbibition). Interestingly, early induction of *OsSAUR33* expression was observed in accessions harboring Hap 2 (high seed vigor) but not in accessions harboring Hap 1 (low seed vigor) during seed germination (Figure 8F,H).

## 3. Discussion

High seed vigor, including rapid, uniform germination and vigorous seedling growth, is essential for direct seeding of rice [5]. Identification and utilization of seed vigor-related genes are important for improving seed vigor in rice [3]. In this study, we identified 48 *OsSAUR* genes based on their typical auxin-inducible domain (pfam02519) in rice. This is fewer *OsSAUR* genes than the 58 *OsSAUR* members identified in a previous study [28] because of the updated data and more stringent criteria used in our study. Several studies have demonstrated that SAURs functions as positive effectors of cell expansion during plant growth [30,31,32,33]; however, their function in seed vigor is not well studied. In this study, we found that the disruption of *OsSAUR33* reduced seed vigor in rice. To the best of our knowledge, this is the first report highlighting the involvement of *SAUR* regulation in seed vigor in rice.

Several studies have revealed that different *SAUR* genes exhibit specific expression patterns throughout plant development in cotton (*Gossypium hirsutum*) [38], maize (*Zea mays*) [39] and *Arabidopsis* [40]. Similarly, we observed that fewer than 15 *OsSAUR* genes were expressed during seed germination in rice. Among these, only *OsSAUR33* exhibited specific expression in both embryo and endosperm tissues during seed germination. Therefore, we focused on the role of *OsSAUR33* in seed vigor in this study. Our results showed that *OsSAUR33* exhibited relatively higher expression at the late seed maturation and the early germination stages. This suggests that *OsSAUR33* might function in regulating seed vigor through influencing seed development and early seed germination in rice. The plasma membrane-localized SAURs have been shown to function in cell elongation in *Arabidopsis* and rice, while a number of cytosol- or nucleus-localized SAURs probably function in cell division [31,34,41,42,43]. In this study, we found that OsSAUR33 likely localized to the plasma membrane and the nucleus. Whether *OsSAUR33* also regulates seed vigor via cell elongation or cell division needs to be investigated in the future.

The plant SnRK1 subfamily is primarily involved in carbohydrate metabolism, starch biosynthesis, fertility, stress responses, seed germination, and seedling growth [26,44]. In this study, we observed that OsSAUR33 interacted with OsSnRK1A in rice, implying that *OsSAUR33* is involved in carbohydrate metabolism, starch biosynthesis, and seed germination. Seed maturation is the most important stage for establishing seed vigor, as soluble sugars such as glucose and fructose progressively disappear, while storage carbohydrates such as starch increase during seed maturation [45]. We thus speculated that OsSAUR33 regulates seed vigor by influencing seed quality due to its high expression at the mature seed stage and because it interacted with OsSnRK1A. A decrease of SnRK1 activity led to an increase of sucrose accumulation at seed maturation in pea (*Pisum sativum*) [46]. Similarly, we observed that the knockout of *OsSAUR33* resulted in higher soluble sugars in the mature grains of rice.

SnRK1s function as sensors to monitor cellular carbohydrate status and/or AMP/ATP levels to maintain the equilibrium of sugar production and consumption necessary for proper growth [26,44,47]. Therefore, we assumed that *OsSAUR33* may regulate seed vigor by influencing the accumulation of sugars during seed maturation and seed germination stages. Rice SnRK1A acts upstream of *OsMYBS1* and *aAmy3* and plays a central role in the sugar signaling pathway by regulating their expression during seed germination [21]. A recent study indicated that *OsMYBS1* promotes *αAmy3* expression under sugar starvation, whereas *OsMYBS2* represses *αAmy3* expression in rice [22]. Therefore, we analyzed the role of *OsSAUR33* in regulating seed vigor by focusing on the sugar signaling pathway. We observed that the knockout of *OsSAUR33* resulted in lower expression of *OsMYBS1* due to the higher soluble sugar accumulation in the early germinating seeds, and the reduced levels of OsMYBS1 then resulted in reduced *aAmy3* expression and α-amylase activity. Moreover, we confirmed exogenous glucose-induced reduction of rice seed vigor in this study. Our data preliminarily demonstrate a positive role of *OsSAUR33* in seed vigor by maintaining the sugar balance during seed maturation to promote *OsMYBS1* and *aAmy3* expression at the early germination stage for hydrolysis of starch. However, the role of the OsSAUR33–OsSnRK1A interaction and whether OsSnRK1A directly interacts with OsMYBS1 in the regulation of seed vigor needs to be further investigated.

We also analyzed the allelic diversity of *OsSAUR33* using 180 randomly selected rice accessions, including *indica* and *japonica* accessions [37]. After analyzing the SNP data of the rice accessions, we identified the Hap 2 haplotype of *OsSAUR33* that positively correlated with seed vigor. Interestingly, the elite Hap 2 haplotype mainly existed in *indica* accessions but not in *japonica* accessions. The early induction of *OsSAUR33* expression was observed in accessions harboring Hap 2 during seed germination. This suggests that the early induction of *OsSAUR33* during seed germination might contribute to seed vigor in rice. However, how the variations of *OsSAUR33* affect its expression pattern and contribute to seed vigor needs to be further investigated. The determination of *OsSAUR33* allelic diversity with a focus on newly identified elite rice accessions is of interest. Indeed, we identified several elite accessions from China harboring Hap 2, including Ai-Chiao-Hong, Pao-Tou-Hung, TeQing, ZHE 733, Zhenshan 2, Chang Ch’Sang Hsu Tao, and Zhenshan 97B. These elite accessions might be useful for improving seed vigor in rice. We speculate that the seed vigor of *japonica* rice could be improved by introducing Hap 2 from *indica* into *japonica* rice accessions. The confirmation of this hypothesis is now in progress.

## 4. Materials and Methods

### 4.1. Plant Materials and Growth Conditions

Two *OsSAUR33* mutants (*ossaur33-1* and *ossaur33-2*) in the *japonica* Nipponbare background (*Oryza sativa* L.) were generated using the CRISPR/Cas9 system. The two mutants were generated using two target guide sequences in the exon of *OsSAUR33* were cloned into the pHUE411 plasmid vector. The mutants were identified by direct sequencing of the PCR products from the editing site using specific primers (Table S6). All plants were grown in an experimental field at South China Agricultural University. Seeds were harvested at maturity stage and dried at 42 °C for 7 days to break seed dormancy [12].

### 4.2. Characterization of the *OsSAUR* Family

The information and sequences of *OsSAURs* were downloaded from the rice genome annotation project (http://rice.plantbiology.msu.edu/). The conserved domains and Pfam searches were performed after removing redundant gene sequences with default parameters (https://www.ncbi.nlm.nih.gov/Structure/cdd/wrpsb.cgi; http://pfam.xfam.org/). Multiple sequence alignment was conducted with ClustalW, which was integrated in Mega v6.0 [48]. Phylogenetic analysis was performed through the online software PhyML 3.0 using the maximum-likelihood method with default parameters [49,50]. The expression of *OsSAURs* in embryo and endosperm tissues during seed germination was investigated using Genevestigator in rice (https://www.genevestigator.com/).

### 4.3. Evaluation of Seed Vigor

The evaluation of seed vigor was conducted according to He et al. [12] under normal and direct seeding conditions. Thirty seeds per replicate of the *ossaur33* mutants and WT Nipponbare were germinated in 9 cm-diameter Petri dishes under normal conditions at 25 ± 1 °C for 9 days. Meanwhile, 30 seeds per replicate were sown in 1 cm-deep soils at 25–30 °C for 9 days. Additionally, the influence of glucose (3% and 6%) treatments on seed vigor was also tested. The criteria for seed germination and seedling establishment were as stated in He et al. [3]. Seed vigor, including germination potential, germination index, germination percentage, time to reach 50% germination, and seedling percentage, were calculated. Three biological replications were performed.

### 4.4. Quantitative Reverse Transcription Polymerase Chain Reaction (qRT-PCR) Analysis

Total RNA was extracted from various tissues (panicle, root, stem, leaf, and internode), the developing grains (0, 7, 14, 21, 28, and 32 days after flowering), and germinating seeds (0, 4, 12, 24, 36, 48, 60, and 72 h of imbibition) of the WT using the HP Plant RNA Kit (Omega, Atlanta, GA, USA) following the manufacturer’s instructions. The qRT-PCR reactions were performed in a CFX96 Real-Time System (Bio-Rad, CA, USA) with the rice *OsActin* as an internal control. The PCR conditions were as follows: 95 °C for 2 min, followed by 40 cycles of 95 °C for 5 s and 60 °C for 10 s. Primers used for qRT-PCR are listed in Supplemental Table S6. Normalized transcript levels were calculated using the comparative C_T_ method [51]. Three biological replications were performed.

### 4.5. β-Glucuronidase (GUS) Staining and Subcellular Localization Assay

Transgenic plants carrying the *OsSAUR33* promoter:*GUS* fusion construct in Nipponbare were used for a GUS staining assay. Briefly, a 2-kb genomic DNA fragment of the 5′ upstream region of *OsSAUR33* was amplified by PCR. These fragments and the GUS gene were cloned into the pCAMBIA 1304 plasmid vector. GUS staining of tissues from the positive transgenic plants was performed as previously described [52]. The open reading frame of *OsSAUR33* (without the stop codon) was amplified and inserted into the *pCambia1305-GFP* vector driven by the CaMV 35S promoter according to the manufacturer’s instructions (Vazyme, Nanjing, China). The plasma membrane (PM) marker RFP:SYP122 and nucleus marker RFP:Fib2 were used for co-localization analysis. Then, the construct was introduced into *Agrobacterium tumefaciens* strain GV3101 and infiltrated into *Nicotiana benthamiana* leaves [53]. The fluorescence signals were then detected by a LSM780 confocal fluorescence microscope (http://www.zeiss.com).

### 4.6. Differentially Expressed Genes Analysis

Total RNA was extracted from WT and *ossaur33-1* seeds after 12 h of imbibition using HP Plant RNA Kit (Omega, Atlanta, GA, USA) according to the manufacturer’s instructions. Construction of cDNA libraries and BGISEQ-500RS sequencing were performed at BGI-Wuhan Co., Ltd., Wuhan, China. Levels of gene expression were quantified in terms of fragments per kilo base of exon per million (FPKM) using RNA-Seq by Expectation-Maximization (RSEM) version 1.1.11 [54]. The DEGs with a *P*-adj (*p*-adjusted) <0.001 and fold change ≥2.0 were selected for further KEGG pathway analysis. Three biological replications were performed.

### 4.7. Sugar Content and Amylase Activity Assays

The dry mature grains of *ossaur33* mutants and WT plants and their seeds after 6, 48, and 72 h of imbibition in 9 cm-diameter Petri dishes at 25 ± 1 °C were harvested to detect the levels of total soluble sugar, glucose, and *α*-amylase activity by using commercial assay kits, according to the manufacturer’s instructions (Suzhou Keming Bioengineering Company, Suzhou, China). Three biological replications were performed.

### 4.8. Bimolecular Fluorescence Complementation (BiFC) and Luciferase (LUC) Assays

The OsSAUR33 and OsSnRK1A were fused with the C-terminus or N-terminus of the split-yellow fluorescent protein (YFP) by homologous recombination, respectively, for the BiFC assay. The vectors of pCAMBIA1300-nLUC and pCAMBIA1300-cLUC were used for LUC assay. Different recombinant plasmid including p2YN-OsSnRK1A, p2YC-OsSAUR33, nLUC-OsSnRK1A and cLUC-OsSAUR33 with the control vector were introduced into *Agrobacterium* strain GV3101. Overnight agrobacteria cultures were resuspended with infiltration buffer (10 mM MgCl_2_, 0.1 mM acetosyringone, and 10 mM MES). Different experiment and control group agrobacteria suspension were mixed and co-infiltrated into 5- to 6-week-old *Nicotiana benthamiana* leaves by using a needleless syringe, then weak light growth. YFP fluorescence was observed by confocal microscopy after two days. Luciferin (1 mM) was sprayed onto the leaves, and the plants were kept in the dark for 2 to 5 min. LUC images were captured using a cooled CCD imaging apparatus [53]. The primers were listed in Supplemental Table S6.

### 4.9. Maltose Binding Protein (MBP) Pull-Down Assay

Clone the full-length cDNA of *OsSAUR33* and *OsSnRK1A* into pGEX-6p-1 and pMAL-c2 vector, respectively. Transform constructs into the *E. coli* strain BL21 (TSV-A09, Tsingke, PRC) to produce the GST-OsSAUR33 and MBP-SnRK1A fusion proteins. For pull-down, 15 μg SnRK1A-MBP or MBP was incubated with MBP-Tag Dextrin Resin (ATSSE0401, Abbkine, USA) at 4 °C for 2 h then 40 μg OsSAUR33-GST was added and incubated overnight at 4 °C. The beads were washed three times with PBS (SL6110, Coolaber, PRC) + 1% Triton 100 (CT11451, Coolaber, PRC) and three times with PBS, then were boiled at 95 °C for 10 min with 5× sodium dodecyl sulfate (SDS) loading buffer (SL1180, Coolaber, PRC). For western blotting, the GST antibody (Cell signaling technology, Danvers, MA, USA) and MBP antibody (Cell signaling technology, Danvers, MA, USA) were used to detected the proteins.

### 4.10. Haplotype Analyses

The 700,000 SNP markers of rice accessions were used to determine the haplotypes of *OsSAUR33* (https://ricediversity.org/) [37]. Haplotype analyses were conducted according to He et al. [12]. The seed vigor of the 180 randomly selected accessions was tested in 9 cm-diameter Petri dishes at 25 ± 1 °C for 9 days (Table S4). The haplotypes represented at least 10 investigated accessions that were considered.

### 4.11. Data Analysis

Experimental data were analyzed using the SAS software (Cary, NC, USA), and significant differences among samples were compared using Student’s *t*-test or analysis of variance (ANOVA) test.

## Supplementary Materials

The following are available online at https://www.mdpi.com/1422-0067/22/4/1562/s1, Figure S1. Characterization of the *OsSAUR* gene family in rice. (A) Phylogenetic relationships of 48 *OsSAUR* genes in rice. The tree was generated using PhyML 3.0 with the maximum-likelihood method. (B,C) Expression pattern of *OsSAUR* genes in embryo and endosperm tissues during 24 h of imbibition using Genevestigator (http://www.genevestigator.com) in rice. Red, up-regulation; gray, no change; blue, down-regulation. Values represent the log_2_ fold changes of genes; Figure S2. The mutants were generated using the clustered regularly interspaced short palindromic repeats (CRISPR)/CRISPR-associated protein 9 (Cas9) system. (A,B) Gene structures of the WT and *ossaur33* mutants. (C) Sequence alignment of the WT and *ossaur33* mutants in editing sites. (D) Comparison of amino acid sequences between the WT and *ossuar33* mutants in rice; Figure S3. Comparison of grain and agronomic traits between WT and *ossaur33* mutants in rice. (A,C) grain length; (B,D) grain width; (E) grain thickness; (F) 1000-grain weight; (G) plant height; (H) heading date; (I) number of tillers/plant; (J) number of grains/main panicle. Bar = 1 cm. Each column represents the mean ± standard deviation, *n* = 10 for grain length, width and thickness, *n* = 4 for 1000-grain weight, plant height, heading date, number of tillers/plant and number of grains/main panicle; Figure S4. The original western blot images of the pull-down assay in vitro; Table S1. Detailed information of the 48 identified *OsSAUR* genes in rice; Table S2. DEGs between WT and *ossaur33-1* seeds after 12 h of imbibition in rice; Table S3. DEGs involved in starch and sucrose metabolism during seed germination in rice; Table S4. Information of the 180 accessions used for haplotype analyses in rice; Table S5. Information and seed vigor of the accessions harboring Hap 1 and Hap 2 of *OsSAUR33* in rice; Table S6. The primer pairs used in this study.
